# Evaluating vaccination timing, hesitancy and effectiveness to prevent future outbreaks: insights from COVID-19 modelling and transmission dynamics

**DOI:** 10.1098/rsos.240833

**Published:** 2024-11-13

**Authors:** Komal Tanwar, Nitesh Kumawat, Jai Prakash Tripathi, Sudipa Chauhan, Anuj Mubayi

**Affiliations:** ^1^ Department of Mathematics, Central University of Rajasthan, Bandar Sindri, Kishangarh 305817, Ajmer, Rajasthan, India; ^2^ Modelling & Simulation, Health Economics and Market Access, Evidera, Ottawa, Canada; ^3^ Kalam Experts, Vishakhapatnam, India; ^4^ Intercollegiate Biomathematics Alliance, Illinois State University, Normal, IL, USA; ^5^ Kalam Institute of Health Technology, Vishakhapatnam, Andhra Pradesh, India

**Keywords:** COVID-19, vaccine hesitancy, vaccine efficacy, social interventions, behaviour compensation

## Abstract

The COVID-19 vaccine has been available in India since January 2021, although many individuals have refused to take the vaccine for various reasons. Vaccination plays a crucial role in disease control by preventing a substantial number of cases and associated disabilities. However, vaccine hesitancy poses a barrier that hinders these efforts. Our article presents a novel approach by proposing a mathematical model for COVID-19 that incorporates vaccine hesitancy, vaccine efficacy and behaviour compensation post-vaccination. The model is calibrated with COVID-19 incidence data for India from 13 February 2021 to 12 January 2022, using the Markov chain Monte Carlo method. The analysis examines the effects of hesitancy and social interventions through a series of practical simulations. The simulation results show that while COVID-19-infected individuals may have natural immunity, vaccination post-recovery is crucial to reduce cases by up to 64.1%. Social interventions, such as face masks and distancing, remain essential to prevent a rise in cases and ensure effective disease control. The model demonstrates that vaccination, combined with continued social interventions, is crucial for effectively reducing COVID-19 cases and preventing future outbreaks. Addressing vaccine hesitancy and maintaining preventive measures are key to successfully controlling the pandemic.

## 1. Introduction

COVID-19, caused by the SARS-CoV-2 virus, rapidly spread worldwide, leading the World Health Organization (WHO) to declare it a pandemic on 11 March 2020. Non-pharmaceutical measures like masks and social distancing were recommended, but experts emphasize vaccination as the best strategy to end the pandemic [1]. India’s first COVID-19 case was reported in Kerala on 30 January 2020, with vaccinations beginning in January 2021. Despite the initial optimism surrounding vaccines [2], individual behaviour and hesitancy have influenced the pandemic’s course, as seen with the second wave even after vaccines became available.

Human behaviour, including actions, reactions, thoughts, emotions and motivations, plays a critical role in epidemiology by influencing disease spread [3]. Protective behaviours like hygiene and social distancing reduce transmission, while risky behaviours, such as attending gatherings or vaccine refusal, increase it [3]. During COVID-19, behaviours like protective actions, misinformation and health-seeking were particularly prominent [4]. Mathematical models have been valuable tools for examining the impact of these behaviours on infectious disease dynamics across various scales [2,3,5–11].

Vaccine hesitancy, a key aspect of human behaviour, influences the transmission of infectious diseases like COVID-19. It can lead to delays in vaccine acceptance or refusal, reducing vaccination coverage and undermining public health efforts. Persistent misinformation, such as claims that vaccines cause infertility, HIV or cancer, fuels this hesitancy [12]. Among parents, concerns about the dangers of immunizing children further drive anti-vaccine beliefs [13]. This hesitancy is not new, as the anti-vaccine movement has existed since vaccines were developed, contributing to disease outbreaks and lower vaccine acceptance rates [13].

Vaccine hesitancy is complex and can be understood through the epidemiological triad, which considers environmental, agent and host factors [14,15]. Environmental factors include public health policies, social influences and media messaging [13,16,17]. The agent (vaccine and pathogen) factors involve the perception of vaccine safety and effectiveness, in addition to the perceived susceptibility to the disease [13,18,19]. Host factors depend on knowledge, previous experience, education and income levels [15,20].

Several researchers have studied COVID-19 vaccine hesitancy, with most relying on statistical models or machine learning techniques using survey data [21–25]. These approaches have been used to assess vaccination readiness and the impact of media on vaccine distrust [22,24]. Studies have also explored how different information sources influence vaccination attitudes [23,25,26]. While survey data analysis is valuable for identifying vaccine hesitancy predictors, it is often limited by data availability and geographic specificity, and it cannot simulate the interaction between disease dynamics and hesitancy [27].

Mathematical modelling offers an alternative, capable of overcoming these limitations by generalizing results across populations and providing a deeper understanding of how vaccine hesitancy and disease dynamics interact. Recent studies have highlighted the importance of comprehensive health measures, like vaccination and masking, in mitigating outbreaks [28]. Other works, such as by Wang *et al*. [29] and RabieiMotlagh *et al*. [30], focus on epidemic dynamics, network topology and the stochastic nature of disease spread. Models that incorporate vaccine hesitancy into disease dynamics have proven useful for predicting outbreaks and guiding public health strategies [31–33]. Liu *et al*. [34] also developed a reaction–diffusion model to examine the impact of latent periods and infection density on disease spread, offering valuable insights for effective control measures.

Reno *et al*. [26] explored the relationship between information sources and COVID-19 vaccine hesitancy, finding that traditional media use reduced hesitancy, while social media use increased it. They suggested that public health messaging should focus on reliable sources to combat hesitancy. Oduro *et al*. [32] developed a mathematical model to examine the impact of vaccine education on COVID-19 containment, showing that education significantly reduced cases and fatalities by increasing vaccine willingness. Despite many empirical studies on vaccine hesitancy [21–25], few models address hesitancy, prompting us to develop a model incorporating COVID-19 vaccine hesitancy.

In the present research, we propose a mathematical model that incorporates vaccine hesitancy, vaccine efficacy and behaviour compensation post-vaccination. This study aims to (i) estimate the level of vaccine hesitancy in India, (ii) investigate the impact of different parameters on $R_{0}$, and (iii) explore how vaccine hesitancy affects the infection load.

The structure of the paper is as follows. Section 2 presents and formulates the mathematical model. Section 3 discusses the model dynamics, including the basic reproduction number, and the local and global stability of the disease-free equilibrium, as well as the epidemic’s final size. Section 4 covers data fitting and parameter estimation. Section 5 provides numerical simulations of the model. Finally, §6 concludes with the study’s findings.

## 2. Mathematical formulation of the model

**Figure 1.**
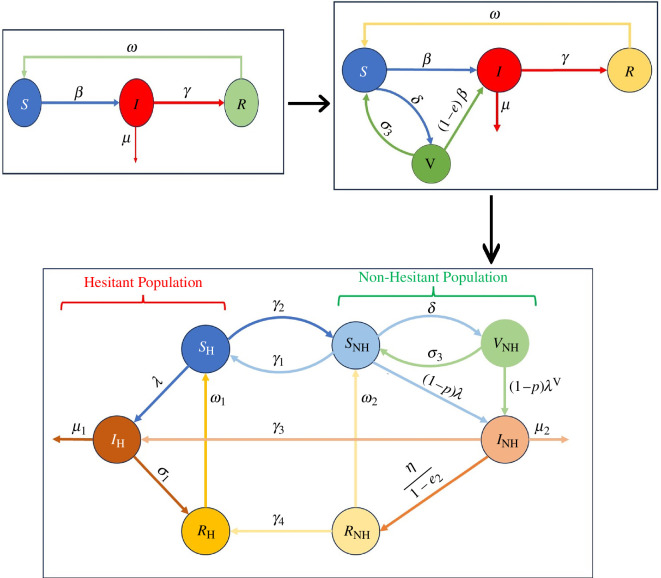
Progression of COVID-19 transmission models. The upper-left diagram depicts the fundamental SIR model, wherein susceptible (S) individuals may become infected (I) and subsequently recover (R). The upper-right diagram expands this concept into the SIRV model, incorporating a vaccinated (V) population and vaccination rate (δ). The lower diagram further extends the model, stratifying the population into hesitant $\left(S_{H},I_{H},R_{H}\right)$ and non-hesitant $\left(S_{NH},I_{NH},R_{NH},V_{NH}\right)$ groups, with additional transitions representing vaccine hesitancy and behavioural dynamics.

The most general and simple SIRS model (figure 1) [35] has been used to model infectious diseases, in which the population is divided into three distinct compartments: susceptible S(t), infected I(t) and recovered R(t). It is assumed that the recovered individuals will again join the susceptible class at the rate ω, owing to waning immunity over time. Susceptible individuals join the infected class at rate β. Infected individuals join the recovered class at a rate of γ. This model can be applied to infectious diseases in which an individual’s immunity may wane over time, such as seasonal influenza and COVID-19. Furthermore, the SIRS model can be extended to the SVIRS model (which includes the vaccination compartment) to study the dynamics of the disease in the presence of a vaccination programme.

In the SVIRS model (figure 1), the assumption has been made that the vaccines are imperfect (i.e. they do not provide 100% protection against disease), so the vaccinated individuals also get infections, but there is a reduction in transmission rate due to the vaccine efficacy, which is considered by the factor (1−e), 0<e<1. After vaccination, susceptible individuals move to the vaccination compartment at rate δ, and vaccinated individuals again join the susceptible class at rate $\sigma_{3}$ because of waning immunity. However, the decision to vaccinate always depends on the individual’s choice. When making decisions, individuals make them according to their perception that the reason we have not been able to eliminate most vaccine-preventable diseases.

In the case of COVID-19 vaccines, there have been a lot of rumours and conspiracy theories regarding vaccine effectiveness, safety, etc., due to which the hesitancy occurs against vaccination. Therefore, in the present study, we introduce the vaccine hesitancy via mathematical modelling. The term ‘vaccine hesitancy’ means that individuals in the population avoid getting vaccinated or want to wait longer. Depending on the hesitancy and non-hesitancy against the vaccination, we introduce two groups: hesitant and non-hesitant individuals against the vaccination in the SVIRS model. Additionally, we divide the total population N(t) at time t into seven distinct compartments, namely hesitant susceptible $S_{H}$, non-hesitant susceptible $S_{NH}$, hesitant infectious $I_{H}$, non-hesitant infectious $I_{NH}$, vaccinated $V_{NH}$, hesitant recovered $R_{H}$ and non-hesitant recovered $R_{NH}$. Thus,


(2.1) $$\begin{array}{ccc} & N(t)=S_{H}(t)+S_{NH}(t)+I_{H}(t)+I_{NH}(t)+V_{NH}(t)+R_{H}(t)+R_{NH}(t). & \end{array}$$

Our proposed model includes the following assumptions:

— The total susceptible population involves both hesitant ($S_{H}$) and non-hesitant population ($S_{NH}$).
— It is assumed that if the individuals from the hesitant susceptible class get the infection, they move to the hesitant infectious class. Similar scenario with the individuals in the non-hesitant susceptible class.
— It is also assumed that individuals from the non-hesitant susceptible class can join the hesitant susceptible class ($S_{NH}$ to $S_{H}$) when the disease spread is less.
— Individuals from the hesitant susceptible class can join the non-hesitant susceptible class ($S_{H}$ to $S_{NH}$) due to fear of the disease when the disease spread is high.
— It is assumed that the non-hesitant individuals are more health conscious and may follow the social intervention norms, such as social distancing, use of face-masks, etc.; consequently, the transmission rate of non-hesitant susceptible individuals reduces as compared with the hesitant susceptible class.
— The model considers that the vaccine is only administered to the non-hesitant susceptible individuals ($S_{NH}$) at a rate, δ.
— The vaccine is assumed to be imperfect (i.e. it does not provide 100% protection against COVID-19). Non-hesitant individuals after vaccination may get infected too at the rate $\lambda^{V}$ but there is a reduction in the susceptibility of the vaccinated individuals to infection acquisition, which is considered via multiplying the transmission rate by a factor ($1-e_{1}$), where $e_{1}$ is the vaccine efficacy and $0<e_{1}<1$. Also, the effect of the vaccine may even wane off and thus vaccinated populations again join non-hesitant susceptible at the rate $\sigma_{3}$.
— There may be an increase in social contact rate like reducing social distancing (behaviour compensation post-vaccination) among those who are vaccinated which makes them prone to catching an infection and it is considered via multiplying the transmission rate by a factor ($1+\theta_{1}$), where $\theta_{1}$ represents the behaviour compensation post-vaccination.
— For the non-hesitant infected population who are vaccinated, there is a reduction in the duration of infection and it is considered via dividing the recovery rate η by a fraction $\left(1-e_{2}\right)$, where $e_{2}$ is the vaccine efficacy that reduces the duration of infection and $0<e_{2}<1$.
— The non-hesitant infected individuals move to hesitant infected individuals ($I_{NH}$ to $I_{H}$) as they might rely on naturally attained immunity.
— The hesitant infected population die at higher rate in comparison with non-hesitant infected individuals, thus $\mu_{1}>\mu_{2}$, due to hesitancy towards vaccination.
— There is movement from non-hesitant recovered population $\left(R_{NH}\right)$ to hesitant recovered $\left(R_{H}\right)$ as they might rely on naturally attained immunity.

As per the above model assumptions, the mathematical model can be written in the following form of nonlinear ordinary differential equations:


(2.2) $$\begin{array}{rl} \frac{dS_{H}}{dt} & =-\lambda S_{H}-\gamma_{2}S_{H}+\omega_{1}R_{H}+\gamma_{1}S_{NH},\\ \frac{dS_{NH}}{dt} & =-\delta S_{NH}-\lambda (1-p)S_{NH}-\gamma_{1}S_{NH}+\gamma_{2}S_{H}+\sigma_{3}V_{NH}+\omega_{2}R_{NH},\\ \frac{dI_{H}}{dt} & =\lambda S_{H}-\sigma_{1}I_{H}-\mu_{1}I_{H}+\gamma_{3}I_{NH},\\ \frac{dI_{NH}}{dt} & =\lambda (1-p)S_{NH}+\lambda^{V}(1-p)V_{NH}-\left(\frac{\eta }{\left(1-e_{2}\right)}+\mu_{2}+\gamma_{3}\right)I_{NH},\\ \frac{dV_{NH}}{dt} & =-\lambda^{V}(1-p)V_{NH}+\delta S_{NH}-\sigma_{3}V_{NH},\\ \frac{dR_{H}}{dt} & =\sigma_{1}I_{H}-\omega_{1}R_{H}+\gamma_{4}R_{NH},\\ \frac{dR_{NH}}{dt} & =\frac{\eta }{\left(1-e_{2}\right)}I_{NH}-\left(\omega_{2}+\gamma_{4}\right)R_{NH}, \end{array}$$

where the force of infection [36] experienced by susceptible individuals is given by


$$\lambda =\frac{\beta (I_{H}+(1-p)I_{NH})}{N},$$

and the force of infection experienced by non-hesitant vaccinated individuals is given by


$$\lambda^{V}=(1-e_{1})(1+\theta_{1})\lambda .$$

The model is subjected to the following non-negative initial conditions: $S_{H}\geq 0,$ $S_{NH}\geq 0,$ $I_{H}\geq 0,$ $I_{NH}\geq 0,$ $V_{NH}\geq 0,$ $R_{H}\geq 0$ and $R_{NH}\geq 0$.

The schematic diagram and biological interpretations of the model parameters are given in figure 1 and table 1, respectively.

**Table 1.** Biological interpretations of the model parameters.

parameters	biological interpretations
p	portion of non-hesitant individuals who are not contacting with the infected individuals due to social interventions and use of face masks
δ	vaccination rate
$\gamma_{1}$	transferring rate from $S_{NH}$ to $S_{H}$ when disease spread is low
$\gamma_{2}$	transferring rate from $S_{H}$ to $S_{NH}$ due to fear of the disease when disease spread is high
$\omega_{1}$	rate at which $R_{H}$ joins $S_{H}$ due to immunity waning over time
$\omega_{2}$	rate at which $R_{NH}$ joins $S_{NH}$ due to immunity waning over time
$\sigma_{1}$	recovery rate of the hesitant infected individuals
$\sigma_{3}$	rate at which vaccinated individuals rejoin the non-hesitant susceptible class due to waning immunity
$\mu_{1}$	disease induced mortality rate of the hesitant infected individuals
$\mu_{2}$	disease induced mortality rate of the non-hesitant infected individuals
β	disease transmission rate
$e_{1}$	vaccine efficacy that reduces the susceptibility of vaccinated individuals
$e_{2}$	vaccine efficacy that reduces the duration of infection
η	recovery rate of non-hesitant infected individuals
$\theta_{1}$	behaviour compensation post-vaccination
$\gamma_{3}$	rate at which non-hesitant infected individuals become hesitant relying on naturally attained immunity
$\gamma_{4}$	rate at which non-hesitant recovered individuals become hesitant relying on naturally attained immunity

## 3. Mathematical results

### 3.1. Non-negativity and boundedness of the model system (2.2)

From the first equation of the model (2.2), we have the following inequality:


$$\begin{array}{r} \frac{dS_{H}}{dt}\geq -\lambda S_{H}-\gamma_{2}S_{H}. \end{array}$$

Using the separation of variable method, we obtain


$$\begin{array}{c} S_{H}(t)\geq S_{H}(0)e^{-(\lambda +\gamma_{2})t}\geq 0\;\text{for all}\;t\geq 0. \end{array}$$

In a similar manner, we can prove the non-negativity of other variables in model (2.2). Further, adding the equations of the model (2.2) yields


(3.1) $$\frac{dN}{dt}=-\mu_{1}I_{H}-\mu_{2}I_{NH}\leq 0,$$

which implies that the total population N(t) is a decreasing function. Using the initial condition $N(0)=N_{0}$, we observe that the solution of the above differential equation is bounded by $N_{0},$ i.e. $N(t)\leq N_{0}$. Hence, all populations are bounded. We can define the following invariant region for the model system (2.2):


(3.2) $$\begin{array}{ccc} & \Lambda =\left\{(S_{H}(t),S_{NH}(t),I_{H}(t),I_{NH}(t),V_{NH}(t),R_{H}(t),R_{NH}(t))\in {\mathbb{R}}_{+}^{7}:N(t)\leq N_{0}\right\}. & \end{array}$$

### 3.2. Basic reproduction number

The model system (2.2) has disease-free equilibrium (DFE), denoted by $E_{0},$ defined as follows:


(3.3) $$\begin{array}{ccc} & E_{0}(S_{H_{0}},S_{NH_{0}},I_{H_{0}},I_{NH_{0}},V_{NH_{0}},R_{H_{0}},R_{NH_{0}})=(S_{H}(0),S_{NH}(0),0,0,V_{NH}(0),0,0), & \end{array}$$

where $S_{H}(0),S_{NH}(0)$ and $V_{NH}(0)$ are the initial population of the hesitant, non-hesitant and vaccinated susceptible individuals. To analyse the asymptotic stability of the DFE, first we compute the basic reproduction number using the method described by Arino *et al*. [37]. Using the notations from [37], consider $x\in {\mathbb{R}}^{2}$ represents the set of infected compartments, $y\in {\mathbb{R}}^{3}$ represents the set of susceptible compartments and $z\in {\mathbb{R}}^{2}$ represents the set of recovered compartments. Therefore $x=(I_{H},I_{NH}{)}^{T}$, $y=(S_{H},S_{NH},V_{NH}{)}^{T}$ and $z=(R_{H},R_{NH}{)}^{T}$. Let *D* be a 3×3 diagonal matrix whose diagonal entries, $\sigma_{i}(i=1,2,3)$, represent the relative susceptibilities of the corresponding susceptible compartments. Let Π be a 2×3 matrix with the property that the (i,j) entry represents the fraction of the jth susceptible compartment that goes into the ith infected compartment upon becoming infected. Also, let b be an n-dimensional row vector of relative horizontal transmission. It follows that the model (2.2) reduces to


(3.4) $$\begin{array}{rl} \dot{x} & =\Pi Dy\beta (x,y,z)bx-Vx,\\ \dot{y} & =-\Pi Dy\beta (x,y,z)bx,\\ \dot{z} & =Wx, \end{array}$$

where W is a 2×2 matrix with the property that the (i,j) entry represents the rate at which individuals of the jth infected compartment move into the ith recovered compartment upon recovery. The 2×2 matrix V describes the transitions between infected compartments as well as removals from infected compartments and is given by


$$V=\left(\begin{array}{cc} \sigma_{1}+\mu_{1} & -\gamma_{3}\\ 0 & \frac{\eta }{\left(1-e_{2}\right)}+\mu_{2}+\gamma_{3} \end{array}\right),$$

and for non-negative vector x, the components of Vx represent the total rate of decrease of each infected compartment. Also


$$b=\left(\begin{array}{cc} 1 & 1-p \end{array}\right),\;\Pi =\left(\begin{array}{ccc} 1 & 0 & 0\\ 0 & 1 & 1 \end{array}\right),\;D=\left(\begin{array}{ccc} 1 & 0 & 0\\ 0 & \left(1-p\right) & 0\\ 0 & 0 & \left(1-p)(1-e_{1})(1+\theta_{1}\right) \end{array}\right).$$

Hence, the reproduction number is given by theorem 2.1 of [37] and expressed as $R_{0}=\beta (0,y_{0},z_{0})bV^{-1}\Pi Dy_{0}$, which yields


(3.5) $$\begin{array}{rl} R_{0} & =\frac{\beta }{\sigma_{1}+\mu_{1}}\frac{S_{H}(0)}{N}+\beta (1-p)[\frac{\gamma_{3}}{\left(\sigma_{1}+\mu_{1})(\frac{\eta }{1-e_{2}}+\mu_{2}+\gamma_{3}\right)}+\frac{\left(1-p\right)}{\frac{\eta }{1-e_{2}}+\mu_{2}+\gamma_{3}}]\frac{S_{NH}(0)}{N}\\ & +\beta (1-p)(1-e_{1})(1+\theta_{1})[\frac{\gamma_{3}}{\left(\sigma_{1}+\mu_{1})(\frac{\eta }{1-e_{2}}+\mu_{2}+\gamma_{3}\right)}+\frac{\left(1-p\right)}{\frac{\eta }{1-e_{2}}+\mu_{2}+\gamma_{3}}]\frac{V_{NH}(0)}{N},\\ & =R_{01}+R_{02}+R_{03}\;(\text{say}). \end{array}$$

Here,


$$\begin{array}{rl} R_{01} & =\frac{\beta }{\sigma_{1}+\mu_{1}}\frac{S_{H}(0)}{N},\\ R_{02} & =\beta (1-p)[\frac{\gamma_{3}}{\left(\sigma_{1}+\mu_{1})(\frac{\eta }{1-e_{2}}+\mu_{2}+\gamma_{3}\right)}+\frac{\left(1-p\right)}{\frac{\eta }{1-e_{2}}+\mu_{2}+\gamma_{3}}]\frac{S_{NH}(0)}{N},\\ R_{03} & =\beta (1-p)(1-e_{1})(1+\theta_{1})[\frac{\gamma_{3}}{\left(\sigma_{1}+\mu_{1})(\frac{\eta }{1-e_{2}}+\mu_{2}+\gamma_{3}\right)}+\frac{\left(1-p\right)}{\frac{\eta }{1-e_{2}}+\mu_{2}+\gamma_{3}}]\frac{V_{NH}(0)}{N}. \end{array}$$

#### 3.2.1. Biological interpretation of *R*_0_

The basic reproduction number $R_{0}$ is the sum of three basic reproduction numbers $R_{01},R_{02}$ and $R_{03}$, where $R_{01}$ represents the average number of secondary infections produced by a hesitant infectious individual into the entire susceptible hesitant population in its average infectious period $1/\sigma_{1}+\mu_{1}$ with the transmission rate β. $R_{02}\;\left(R_{03}\right)$ represents the sum of the average number of secondary infections produced by a non-hesitant infectious individual into the entire non-hesitant susceptible (vaccinated) population in its average infectious period $\frac{1}{\frac{\eta }{1-e_{2}}+\mu_{2}+\gamma_{3}}$ with the transmission rate $\beta (1-p{)}^{2}(\beta (1-p{)}^{2}(1-e_{1})(1+\theta_{1}))$ and the average number of secondary infections produced by a hesitant infectious individual into the entire susceptible (vaccinated) non-hesitant population in its average infectious period $1/\sigma_{1}+\mu_{1}$ with the transmission rate $\beta (1-p)(\beta (1-p)(1-e_{1})(1+\theta_{1}))$, where $\left[\frac{\gamma_{3}}{\frac{\eta }{1-e_{2}}+\mu_{2}+\gamma_{3}}\right]$ represents the proportion of non-hesitant infectious individuals that survived in the non-hesitant infectious compartment and move to the hesitant infectious compartment. It should be noted that the computation of $R_{0}$ from theorem 2.1 of [37] also implies the local asymptotic stability of the disease-free equilibrium $E_{0}$ for the model (2.2). Furthermore, for global asymptotic stability, we have the following result.

**Theorem 3.1**. *The disease-free equilibrium* $E_{0}$ *of the model system* (2.2) *is globally asymptotically stable when* $R_{0}\leq 1$.

*Proof.* The proof is given in appendix A.1.∎

### 3.3. Computation of the final size relations of the epidemic

In this section, we compute the final size of the pandemic using the approach given in Arino *et al*. [37], which are natural quantities associated with the dynamics of epidemic models (with no vital/demographic dynamics), allowing for the realistic quantification of disease burden and can be used to assess the impact and effectiveness of various intervention and mitigation strategies. Using the notation from the previous section, it is convenient to define a three-dimensional vector $\Gamma =[\Gamma_{1},\Gamma_{2},\Gamma_{3}]=\beta bV^{-1}\Pi D$, which yields


$$\begin{array}{ll} \Gamma_{1} & =\frac{\beta }{\left(\sigma_{1}+\mu_{1}\right)},\;\Gamma_{1}=\beta (1-p)[\frac{\gamma_{3}}{\left(\sigma_{1}+\mu_{1})(\frac{\eta }{1-e_{2}}+\mu_{2}+\gamma_{3}\right)}+\frac{\left(1-p\right)}{\frac{\eta }{1-e_{2}}+\mu_{2}+\gamma_{3}}],\\ \Gamma_{3} & =\beta (1-p)(1-e_{1})(1+\theta_{1})[\frac{\gamma_{3}}{\left(\sigma_{1}+\mu_{1})(\frac{\eta }{1-e_{2}}+\mu_{2}+\gamma_{3}\right)}+\frac{\left(1-p\right)}{\frac{\eta }{1-e_{2}}+\mu_{2}+\gamma_{3}}], \end{array}$$

where $V^{-1}=\left(\begin{array}{cc} \frac{1}{\sigma_{1}+\mu_{1}} & \frac{\gamma_{3}}{\left(\sigma_{1}+\mu_{1})(\frac{\eta }{1-e_{2}}+\mu_{2}+\gamma_{3}\right)}\\ 0 & \frac{1}{\left(\frac{\eta }{1-e_{2}}+\mu_{2}+\gamma_{3}\right)} \end{array}\right).$

Furthermore, the final size relations of model (2.2) (or, equivalently, (3.4)), can be established using theorem 5.1 of [37] as follows.

**Theorem 3.2**. *Consider the epidemic model* (2.2) (*or, equivalently,* (*3.4*))*. The final size relations are given by*


$$\begin{array}{rl} \ln\;(\frac{S_{H}(0)}{S_{H}(\infty )}) & \geq \Gamma_{1}D^{-1}\left(\begin{array}{c} \frac{S_{H}(0)-S_{H}(\infty )}{N}\\ \frac{S_{NH}(0)-S_{NH}(\infty )}{N}\\ \frac{V_{NH}(0)-V_{NH}(\infty )}{N} \end{array}\right)+\beta bV^{-1}\left(\begin{array}{c} \frac{I_{H}(0)}{N}\\ \frac{I_{NH}(0)}{N} \end{array}\right),\\ \ln\;(\frac{S_{H}(0)}{S_{H}(\infty )}) & \geq \left(\begin{array}{ccc} \Gamma_{1} & \frac{\Gamma_{2}}{1-p} & \frac{\Gamma_{3}}{\left(1-e_{1})(1-p)(1+\theta_{1}\right)} \end{array}\right)\left(\begin{array}{c} \frac{S_{H}(0)-S_{H}(\infty )}{N}\\ \frac{S_{NH}(0)-S_{NH}(\infty )}{N}\\ \frac{V_{NH}(0)-V_{NH}(\infty )}{N} \end{array}\right)\\ & +\beta \left(\begin{array}{cc} 1 & 1-p \end{array}\right)V^{-1}\left(\begin{array}{c} \frac{I_{H}(0)}{N}\\ \frac{I_{NH}(0)}{N} \end{array}\right),\\ \ln\;(\frac{S_{H}(0)}{S_{H}(\infty )}) & \geq \left(\begin{array}{c} \frac{\Gamma_{1}(S_{H}(0)-S_{H}(\infty )}{N}+\frac{\Gamma_{2}(S_{NH}(0)-S_{NH}(\infty )}{(1-p)N}+\frac{\Gamma_{3}(V_{NH}(0)-V_{NH}(\infty )}{(1-e_{1})(1-p)(1+\theta_{1})N} \end{array}\right)\\ & +\left(\begin{array}{cc} \frac{\beta }{\sigma_{1}+\mu_{1}} & \frac{\beta \gamma_{3}}{\left(\sigma_{1}+\mu_{1})(\frac{\eta }{1-e_{2}}+\mu_{2}+\gamma_{3}\right)}+\frac{\beta (1-p)}{\left(\frac{\eta }{1-e_{2}}+\mu_{2}+\gamma_{3}\right)} \end{array}\right)\left(\begin{array}{c} \frac{I_{H}(0)}{N}\\ \frac{I_{NH}(0)}{N} \end{array}\right),\\ \ln\;(\frac{S_{H}(0)}{S_{H}(\infty )}) & \geq K_{1}[S_{H}(0)-S_{H}(\infty )]+K_{2}[S_{NH}(0)-S_{NH}(\infty )]+K_{3}[V_{NH}(0)-V_{NH}(\infty )]\\ & +K_{4}I_{H}(0)+K_{5}I_{NH}(0),\\ S_{NH}(\infty ) & \geq S_{NH}(0)(\frac{S_{H}(\infty )}{S_{H}(0)}{)}^{\left(1-p\right)},\\ V_{NH}(\infty ) & \geq V_{NH}(0)(\frac{S_{H}(\infty )}{S_{H}(0)}{)}^{\left(1-p)(1-e_{1})(1+\theta_{1}\right)}, \end{array}$$

*where*


$$\begin{array}{rl} K_{1} & =\frac{\beta }{N(\sigma_{1}+\mu_{1})}=K_{4},\\ K_{2} & =\frac{\beta }{N}[\frac{\gamma_{3}}{\left(\sigma_{1}+\mu_{1})(\frac{\eta }{1-e_{2}}+\mu_{2}+\gamma_{3}\right)}+\frac{\left(1-p\right)}{\frac{\eta }{1-e_{2}}+\mu_{2}+\gamma_{3}}]=K_{3}=K_{5}. \end{array}$$

*The conclusion drawn from the final size relation suggests that we can establish an upper limit on the number of individuals who will still be vulnerable once the pandemic concludes by essentially determining a limit on the value of* $S_{H}(\infty )$*. As the initial count of susceptible individuals* $\left(S_{H}(0)\right)$ *is known prior to the onset of the epidemic, we can derive an upper estimate for the anticipated number of people who will contract the disease throughout the epidemic by calculating the difference between* $S_{H}(0)$ *and* $S_{H}(\infty )$*. It is worth emphasizing that in a special instance of the model system (**2.2**), where disease-induced mortality is minimal (e.g.* $\mu_{1}=\mu_{2}=0$*), the inequality within theorem 3.2 transitions to a state of equality, as elucidated by Brauer [**38**]. Consequently, in this particular scenario (where the model system (2.2) employs mass action incidence instead of standard incidence for infection rates), it is possible to calculate precise figures, rather than just the upper and lower limits, for the projected final size of the COVID-19 pandemic. It is worth highlighting that the assumption of negligible disease-induced mortality may not align with reality, especially in the case of diseases such as COVID-19, in which a significant portion of the population experiences substantial mortality.*

## 4. Parameter estimation

The proposed model (2.2) has several parameters, and the values of some of these parameters are known from the literature, as listed in table 2. However, the values of a few parameters were unknown. The unknown parameters are the transmission rate (β), transferring rate ($\gamma_{1}$) from $S_{NH}$ to $S_{H}$ when the disease spread is less, the transfer rate ($\gamma_{2}$) from $S_{H}$ to $S_{NH}$ due to fear of the disease when the disease spread is high, the rate at which non-hesitant infected individuals become hesitant relying on naturally attained immunity $\left(\gamma_{3}\right)$, the rate at which non-hesitant recovered individuals become hesitant relying on naturally attained immunity $\left(\gamma_{4}\right)$, and the parameter $\theta_{1}$ which is behaviour compensation post-vaccination. We estimated these parameters of model (2.2) for COVID-19 data in India from 13 February 2021 to 12 January 2022. The data have been collected for the period spanning from the commencement of the second dose of the COVID-19 vaccine on 13 February 2021 to 12 January 2022. This time frame was selected because no booster doses were administered after the second dose during this period. Our analysis focused on assessing hesitancy in receiving the second dose of the vaccine within this specified duration. We estimate the parameters for the different time periods by dividing the COVID-19 data into distinct stages which allows for capturing finer patterns and gaining a systematic understanding of the pandemic’s progression in India. It also highlights the impact of various public health interventions. Each stage represents a phase in the pandemic with specific challenges and corresponding policy responses, reflecting the evolving nature of the crisis from early surges to strategic mitigation efforts.

**Table 2.** Fixed parameter values with respect to COVID-19 cases in India.

parameters	values	range	reference
p	0.35	0–1	assumed
δ	0.01		[39]
$\omega_{1}$	0.011		[40]
$\omega_{2}$	0.009		[40]
$\sigma_{1}$	0.09	0.015–0.2	[41]
$\sigma_{3}$	0.0055		[42]
$\mu_{1}$	0.0025		[43]
$\mu_{2}$	0.0016		[43]
$e_{1}$	0.7	0–1	[44]
$e_{2}$	0.6	0–1	[44]
η	0.09	0.015–0.2	[41]

During the first stage (13 February 2021 to 8 May 2021), India saw a sharp rise in COVID-19 cases due to the Delta variant, worsened by large gatherings like the Kumbh Mela [45]. The healthcare system faced critical shortages of beds, oxygen and other resources. In response, the government expanded its vaccination campaign, initially targeting healthcare workers and frontline staff, and later including all adults over 18 by 1 May 2021 [46]. This surge in cases prompted a significant increase in vaccination uptake as people became more concerned about the rising infections. During stage 2 (9 May 2021 to 28 December 2021), strict lockdown measures and an accelerated vaccination programme led to a sharp decrease in COVID-19 cases. The government emphasized extensive testing, contact tracing and genomic sequencing to control the virus and identify new variants. However, as cases began to decline, vaccine hesitancy started to emerge, with fewer people feeling the immediate need to get vaccinated. In November and December 2021, a phased reopening of offices and businesses began for a gradual resumption of economic activities [47]. In the third stage (29 December 2021 to 12 January 2022), the emergence of the Omicron variant and the reopening triggered a third wave of infections, characterized by high transmissibility, leading to a surge in cases. This renewed spike in cases once again increased public motivation to get vaccinated, particularly with the introduction of booster doses for high-risk groups starting in January 2022 [48]. The Indian government responded with enhanced preparedness measures such as travel restrictions and booster doses, reflecting the fluctuating public response to the evolving pandemic. The model has been fitted with data taken from the official website of the WHO.

We employed the Markov chain Monte Carlo (MCMC) method, utilizing an adaptive Metropolis–Hastings (M–H) algorithm [49–51] to effectively fit our model. Based on 1000 sample runs, we determined the parameter values for various time intervals. The computed results are summarized in table 3, providing essential statistical metrics for each parameter. The average values depict the central tendencies of the posterior distribution, and serve as representative estimates. Concurrently, standard deviation offers insights into the dispersion of these estimates and provides a measure of variability. To assess convergence, we used the Geweke diagnostic, which compares the means of two subsequences of the chain, assuming the chain has reached stationarity. This method is sensitive to the choice of spectral window and requires subjective judgement [52]. It also assumes the subsequences are asymptotically independent, which can be difficult to validate. Additionally, the Geweke diagnostic evaluates each parameter individually and lacks a multivariate approach, complicating interpretation if only some parameters converge. To address these limitations and provide a more robust assessment, we also used the Gelman–Rubin diagnostic [53]. This widely recognized method is effective for evaluating MCMC convergence. According to Gelman *et al*. [54], if the PSRF (potential scale reduction factor) value for all parameters is close to 1 (less than 1.1), the MCMC chains are considered convergent. Our results, shown in table 3, indicate PSRF values close to 1 for all estimating parameters, suggesting satisfactory convergence. By combining these two diagnostic tests, we can conclude that the MCMC chains have converged and the parameter estimates are reliable. Additionally, we reported 95% confidence intervals (CIs), which delineate the range within which the true parameter values are likely to fall, providing insight into the precision of our estimates. The combination of the DRAM algorithm, MCMC simulations and subsequent statistical analysis offers a comprehensive understanding of the key parameters governing the dynamics of the epidemic [51].

**Table 3.** Parameter estimation by MCMC method.

duration	parameters	mean	standard deviation	Geweke	Gelman–Rubin	95% CI
13 February 2021 to 8 May 2021	$\gamma_{1}$	0.0172	0.0021	0.8054	1.0019	[0.0133–0.0204]
	$\gamma_{2}$	0.0383	0.00841	0.6890	1.0004	[0.0259–0.0554]
	$\gamma_{3}$	0.0064	0.0038	0.2382	1.0018	[0.0006–0.0138]
	$\gamma_{4}$	0.1338	0.0239	0.6649	1.0010	[0.1033–0.1780]
	β	0.3129	0.0162	0.9254	1.0005	[0.2889–0.3457]
	$\theta_{1}$	0.4605	0.2782	0.1895	1.0014	[0.0208–0.8603]
9 May 2021 to 28 December 2021	$\gamma_{1}$	0.0249	0.0153	0.6005	1.0004	[0.0006–0.0624]
	$\gamma_{2}$	0.3943	0.166	0.4713	1.0002	[0.1806–0.7960]
	$\gamma_{3}$	0.1096	0.0303	0.8297	1.0026	[0.0495–0.1711]
	$\gamma_{4}$	0.0543	0.0250	0.6603	1.0002	[0.0090–0.1047]
	β	0.1578	0.0074	0.9253	1.0002	[0.1457–0.1717]
	$\theta_{1}$	0.3174	0.1137	0.6360	1.0001	[0.1620–0.5808]
29 December 2021 to 12 January 2022	$\gamma_{1}$	0.0219	0.0059	0.7559	1.0007	[0.0131–0.0336]
	$\gamma_{2}$	0.0429	0.0290	0.0830	1.0001	[0.0040–0.1128]
	$\gamma_{3}$	0.3200	0.1169	0.8399	1.0009	[0.0786–0.4954]
	$\gamma_{4}$	0.1450	0.0548	0.9129	1.0043	[0.0385–0.2667]
	β	0.3935	0.0226	0.8101	1.0023	[0.3569–0.4414]
	$\theta_{1}$	0.0932	0.0141	0.9487	1.0004	[0.0615–0.1199]

**Figure 2.**
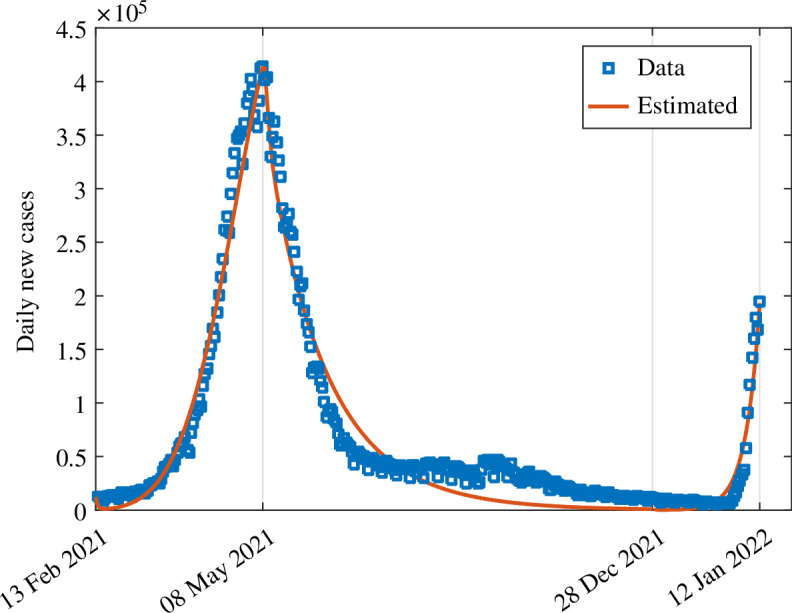
Data fitting of the model system (2.2), showing the model’s output for the daily new cases (orange curve) compared with the observed daily new cases for India (blue dots) from 13 February 2021 to 12 January 2022.

**Figure 3.**
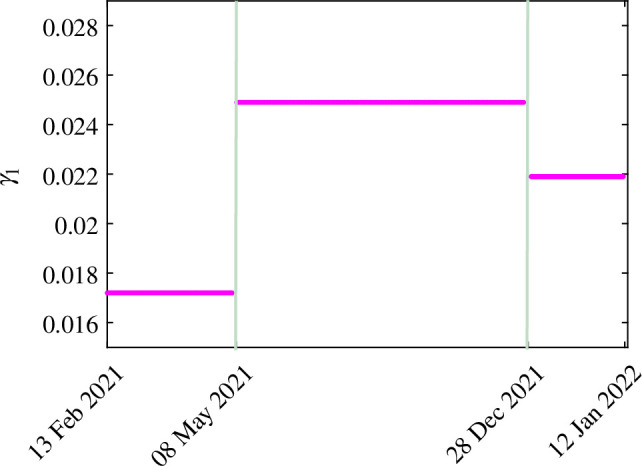
The estimated values of model parameter $\gamma_{1}$ for different time periods.

**Figure 4.**
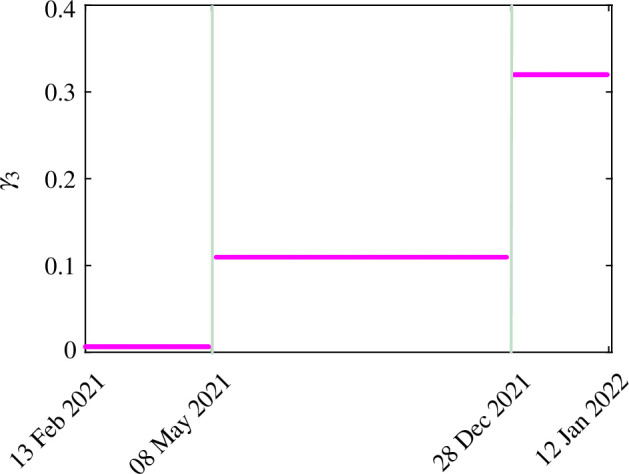
The estimated values of model parameter $\gamma_{3}$ for different time periods.

**Figure 5.**
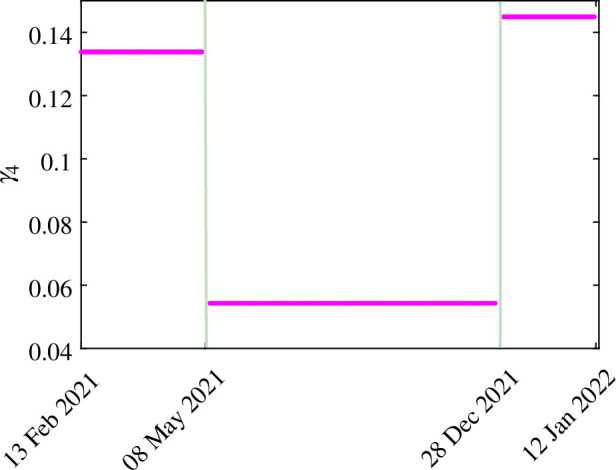
The estimated values of model parameter $\gamma_{4}$ for different time periods.

The results obtained by fitting daily new COVID-19 cases in India are shown in figure 2. The blue curve in figure 2 shows the real data of COVID-19 cases, and the orange curve depicts the model output. The estimated parameters are listed in table 3. The patterns of the estimated hesitancy parameters $\left(\gamma_{1},\gamma_{3},\gamma_{4}\right)$ are shown in figures 3–5.

The known parameters obtained from the literature are listed in table 2. WHO has approved vaccines against COVID-19 with different efficacies [44]. Therefore, we consider $e_{1}=0.7$ (70% vaccine efficacy) as the vaccine efficacy that reduces the susceptibility of vaccinated individuals and $e_{2}=0.6$ (60% vaccine efficacy) as the vaccine efficacy that reduces the duration of infection [44]. Furthermore, we assume that only non-hesitant individuals follow social interventions (social distancing, use of a face mask, etc.), so there has been a reduction in transmission. We set p=0.35, which means that only 65% of non-hesitant individuals contribute to transmitting the disease. After infection, individuals who recover from disease attain natural immunity. The natural immunity attained after COVID-19 recovery varies from person to person and lasts for at least 3 months [40]. The immunity after two doses of the vaccine lasts for at least six months [42]. We consider $\omega_{1}=1/90$ per day as the rate at which the natural immunity of hesitant individuals wanes off, $\omega_{2}=1/111$ per day as the rate at which the natural immunity of non-hesitant individuals wanes off because non-hesitant individuals have some extra immunity due to the vaccine, and $\sigma_{3}=1/180$ per day as the rate at which the vaccinated individuals join the non-hesitant susceptible class due to waning immunity.

### 4.1. Pattern of estimated hesitancy parameters

Individuals showed rational behaviour regarding COVID-19 vaccines. They change their decision according to the situation of the pandemic as to whether they should be vaccinated. The hesitancy parameters $\gamma_{1}$, $\gamma_{3}$ and $\gamma_{4}$ have been estimated for different periods, and it has been observed that the rates are different for the time intervals, as shown in figures 3–5. The estimated value of parameter $\gamma_{1}$ (the transfer rate from $S_{NH}$ to $S_{H}$ when the disease spread is less) from 13 February 2021 to 8 May 2021 was 0.0172 when the number of cases increased. However, from 9 May 2021 to 28 December 2021, people were more hesitant because in this period, the disease spread was less and the estimated value of the hesitancy parameter $\gamma_{1}$ was 0.0249, which is an increase of 44.7% from the hesitancy value of 0.0172. From 29 December 2021 to 12 January 2022, the hesitancy parameter $\gamma_{1}$ decreases and becomes 0.0219 because of the increment in the daily number of cases because people may be afraid of the disease and think that they must be vaccinated.

The hesitancy parameter $\gamma_{3}$ (the rate at which non-hesitant infected individuals become hesitant relying on naturally attained immunity) has not shown a particular pattern for hesitancy and is increasing continuously. The reason for this might be that the infected individuals are not eligible for the vaccine during their infectious period, so their behaviour cannot be determined on the basis of the pandemic. They may behave in accordance with their health status. The hesitancy parameter $\gamma_{4}$ (the rate at which non-hesitant recovered individuals become hesitant relying on naturally attained immunity) shows a distinct pattern in different time periods. Those who recovered from the infection were eligible for vaccination after 14 days. They can opt for vaccination or rely on natural immunity. Hence, they may change their behaviour according to the pandemic situation or their personal preferences. For example, we can observe from figure 5 that from 13 February 2021 to 8 May 2021, the parameter $\gamma_{4}$ has a value of 0.1338, but from 9 May 2021 to 28 December 2021, when the number of cases decreased, the parameter $\gamma_{4}$ has a value of 0.1578, which means that people become less hesitant and opt for the vaccination choice. From 29 December 2021 to 12 January 2022, the value of the hesitancy parameter $\gamma_{4}$ is 0.1450, which is 8% less than 0.1578. Thus, the decisions of recovered individuals depend on their personal choice, as they have naturally attained immunity as an option.

## 5. Numerical simulations

We simulate model (2.2) by taking the baseline parameters from tables 2 and 3 to assess the impact of different strategies on COVID-19 infection in India. By considering the baseline values of these parameters of the model system (2.2), it is worth mentioning that the reproduction numbers for the different periods discussed in table 3, that is, the first, second and third periods, were 3.36, 1.22 and 1.97, respectively.

### 5.1. Impact of the different parameters on $R_{0}$

**Figure 6.**
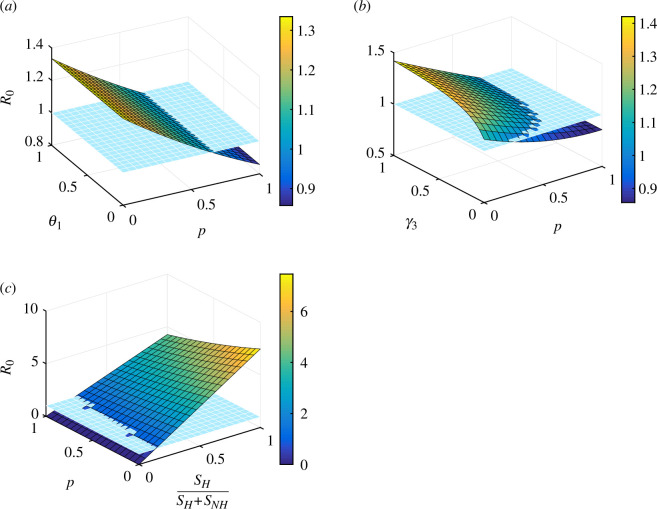
Surface plots illustrate the effects of different parameter combinations on $R_{0}$ as a function of (*a*) p and $\theta_{1}$, (*b*) p and $\gamma_{3}$ and (*c*) p and $\frac{S_{H}}{S_{H}+S_{NH}}$.

Figure 6*a* shows the influence of p and $\theta_{1}$ on $R_{0}$. It is clear from figure 6*a* that the value of p has a greater impact on $R_{0}$ than $\theta_{1}$, which represents behavioural compensation post-vaccination. The simulation results indicate that, if a larger number of individuals adhere to social norms, it will be possible to reduce $R_{0}$ below 1. In figure 6*b*, the influences of parameters p and $\gamma_{3}$ on $R_{0}$ are evaluated. It can be inferred from figure 6*b* that as $\gamma_{3}$ decreases and p increases, $R_{0}$ also decreases. Consequently, it can be concluded from this finding that even after recovering from COVID-19 and acquiring natural immunity, it is important for individuals to receive vaccination and adhere to social norms. In figure 6*c*, we investigate the impact of parameter p and hesitant susceptibility ratio $S_{H}/S_{H}+S_{NH}$ on $R_{0}$. The findings indicate that the influence of the hesitant susceptibility ratio $S_{H}/S_{H}+S_{NH}$ is more significant than that of parameter p on $R_{0}$. This suggests that if the proportion of individuals who are hesitant towards vaccines decreases, which means more people become non-hesitant, then we could reduce the basic reproduction number ($R_{0}$) below one.

### 5.2. Impact of the parameter $\gamma_{1}$ on the infection load

**Figure 7.**
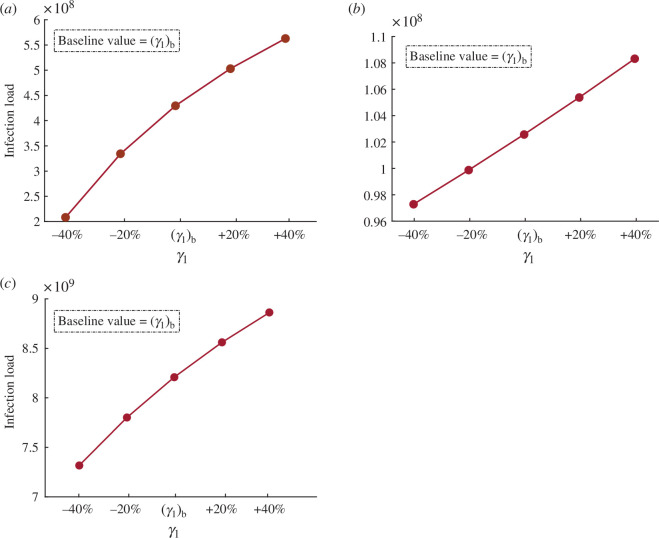
The simulation of the model system (2.2) to assess the impact of the parameter $\gamma_{1}$ on infection load for different time periods. (*a*) From 13 February 2021 to 8 May 2021. (*b*) From 9 May 2021 to 28 December 2021. (*c*) From 29 December 2021 to 12 January 2022.

The simulation results presented in figure 7 indicate that hesitancy towards the COVID-19 vaccine plays a vital role in the rise of COVID-19 cases in India. We used the baseline values of the parameter given in table 3. As shown in figure 7, there seems to be a significant relationship between vaccine hesitancy and infection load. Specifically, as vaccination hesitancy increases, so does infection load. Furthermore, the data suggest that delaying vaccination among those susceptible to infection leads to a subsequent increase in the infection rate. These findings suggest that it is crucial to take proactive measures to address vaccine hesitancy and ensure that vaccinations are administered in a timely manner to minimize the overall burden of infection.

Further, for quantitative results, see appendix A.2. Figure 13*a* illustrates the effect of parameter $\gamma_{1}$ (transferring rate from $S_{NH}$ to $S_{H}$ when the disease spread is less) for the first period (13 February 2021 to 8 May 2021), during which the number of cases increased. The results show that if $\gamma_{1}$ is increased by 20% and 40% from the baseline value, the number of infected individuals increases by 29.17% and 56%, respectively. However, when the value of $\gamma_{1}$ decreased by 20% and 40%, the number of infected individuals decreased by 31.13% and 62.5%, respectively. Figure 13*b* illustrates the effect of parameter $\gamma_{1}$ for the second period (9 May 2021 to 28 December 2021), during which the number of cases decreased. The results show that if $\gamma_{1}$ is increased by 20% and 40% from the baseline value, the number of infected individuals increases by 15.3% and 31.1%, respectively. However, when the value of $\gamma_{1}$ decreased by 20% and 40%, the number of infected individuals decreased by 14.6% and 28.4%, respectively. Similarly, figure 13*c* shows the impact of $\gamma_{1}$ for the third period (29 December 2021 to 12 January 2022) on the infected individuals. The results show that if $\gamma_{1}$ is increased by 20% and 40% from the baseline value, the number of infected individuals increases by 8.7% and 16.5%, respectively. However, when the value of $\gamma_{1}$ decreased by 20% and 40%, the number of infected individuals decreased by 9.9% and 21.2%, respectively.

We can conclude that as the incidence of cases increases, it is highly effective to alleviate the burden of the disease by encouraging more individuals who are susceptible to overcoming their hesitancy to receive the vaccine, thereby increasing the vaccination rate. We would also say that a decrease in reported cases does not necessarily imply a reduction in the overall disease burden, particularly when vaccine hesitancy remains prevalent. Although the number of cases may decline, the burden on the population continues to exist. Therefore, it is essential for individuals to modify their behaviour towards vaccination. Overcoming vaccine hesitancy and embracing vaccination, especially in the face of evolving circumstances, is critical for effectively mitigating the disease burden.

### 5.3. Impact of the parameter $\gamma_{3}$ on infection load

**Figure 8.**
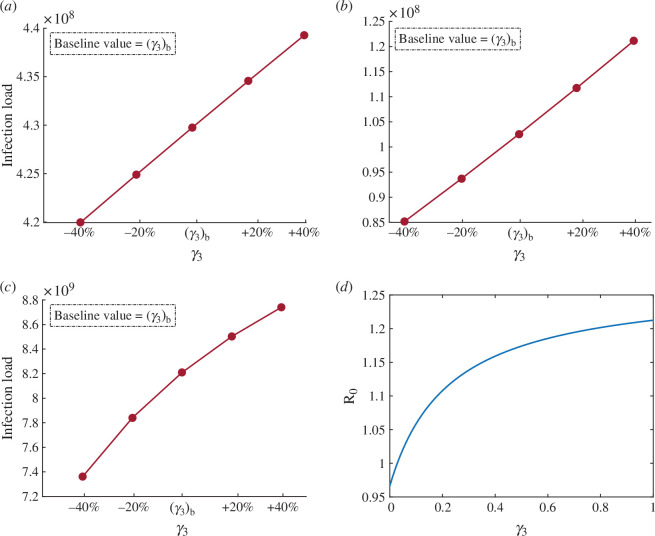
The simulation of the model system (2.2) to assess the impact of the parameter $\gamma_{3}$ on infection load for different time periods. (*a*) From 13 February 2021 to 8 May 2021. (*b*) From 9 May 2021 to 28 December 2021. (*c*) From 29 December 2021 to 12 January 2022. (*d*) Variation of $R_{0}$ with respect to parameter $\gamma_{3}$.

Figure 8 shows the impact of parameter $\gamma_{3}$ (transferring rate from $I_{NH}$ to $I_{H}$ relying on naturally attained immunity) on the infection load. The baseline values are obtained from table 3. From figure 8, it is evident that an increase in $\gamma_{3}$ results in a corresponding increase in infection load. Hence, while COVID-19-infected individuals may possess natural immunity, it is imperative that they receive vaccination post-recovery to mitigate the disease burden. This situation may be further aggravated by any kind of hesitation on their part, leading to a higher disease burden. The quantitative results are presented in appendix A.3. Figure 14*a* depicts the impact of $\gamma_{3}$ when the number of cases increases. From the result, we can say that if we increase $\gamma_{3}$ by 20% and 40% from the baseline value, then the number of infected individuals would increase by 1.52% and 3.03%, respectively. However, the value of $\gamma_{3}$ would decrease by 20% and 40%, and the infected individuals would decrease by 1.53% and 3.04%, respectively. Figure 14*b* depicts the impact of $\gamma_{3}$ for the duration in which the number of cases decreased. From the result, we can say that if we increase $\gamma_{3}$ by 20% and 40% from the baseline value, then the number of infected individuals would increase by 39.9% and 79.4%, respectively. However, when the value of $\gamma_{3}$ decreases by 20% and 40%, the number of infected individuals decreases by 37.4% and 64.1%, respectively.

Figure 14*c* depicts the impact of $\gamma_{3}$ for the duration in which the number of cases decreased. If we increase $\gamma_{3}$ by 20% and 40% from the baseline value, then the infected individuals would increase by 5.8% and 10.8%, respectively. However, when the value of $\gamma_{3}$ decreases by 20% and 40%, the infected individuals decrease by 7.2% and 16.4%, respectively. Furthermore, in figure 8*d*, we show the variation in $R_{0}$ with respect to the hesitancy parameter $\gamma_{3}$.

The study findings imply that individuals who exhibit hesitancy towards vaccination are at an increased risk of contributing to an increase in overall case burden. It is crucial for individuals to recognize that relying solely on natural immunity during periods of declining cases can lead to a false sense of security. According to figure 14*b*, such decisions can result in a resurgence in case numbers. Therefore, it is recommended that individuals, including those with natural immunity, proactively receive a vaccine to prevent the potential escalation of case burdens and maintain public health, even during periods of perceived decline in reported cases. A proactive approach is essential for ensuring continued protection against infectious diseases.

### 5.4. Combined impact of the vaccination rate δ and hesitancy towards vaccination γ

**Figure 9.**
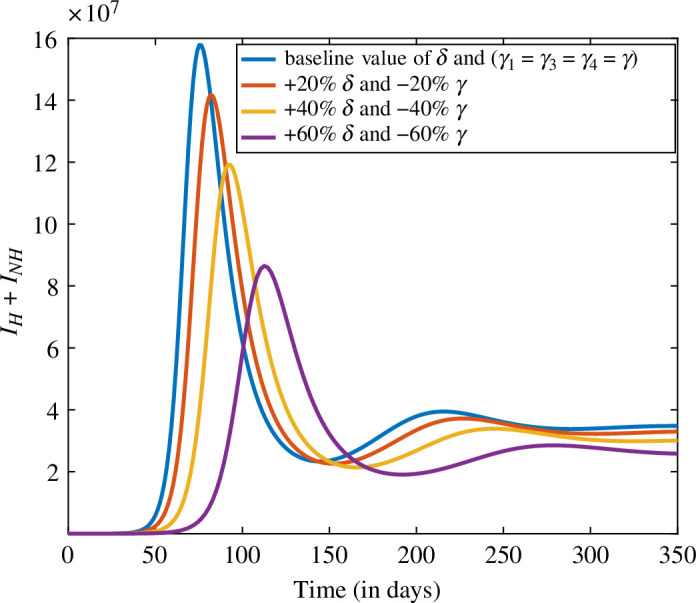
The trend of infected individuals $\left(I_{H}+I_{NH}\right)$ for distinct values of δ and γ.

The present study aims to simulate a time series of infected individuals in India by incorporating varying levels of the vaccination rate (δ) and decreasing levels of the hesitancy rate (γ). For this analysis, we assume that $\gamma_{1}=\gamma_{3}=\gamma_{4}=\gamma$ to assess the effect of hesitancy on the spread of infection. The baseline values of the parameters are sourced from tables 2 and 3 for the period from 28 December 2021 to 12 January, 2022, with the aim of examining the impact of these combined factors on the spread of the disease. The data in figure 9 reveal that increasing the vaccination rate by 20% and reducing the hesitancy rate by 20% can result in a 19.2% decrease in the number of infected individuals. If the same scenario is repeated with increases of 40% and 60% in the vaccination rate, the number of infected individuals decreases by 43.3% and 73.2%, respectively. These findings demonstrate that increasing the vaccination rate while decreasing hesitancy can effectively reduce disease burden.

### 5.5. Combined impact of the parameters p and $\theta_{1}$

**Figure 10.**
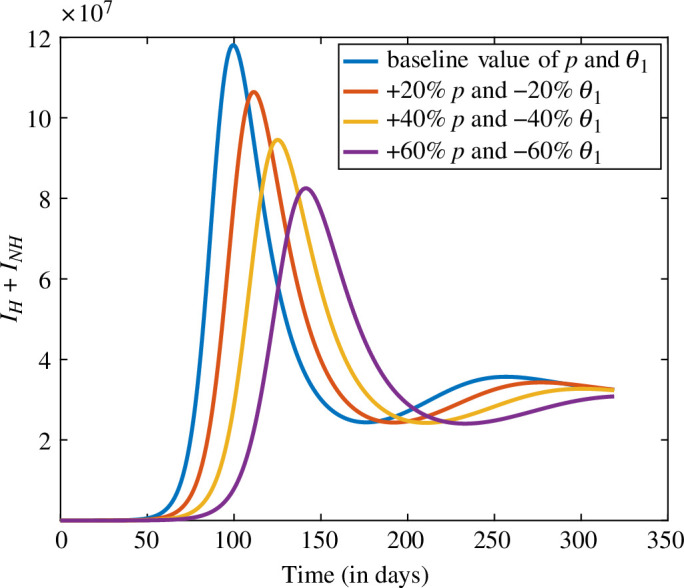
The trend of infected individuals $\left(I_{H}+I_{NH}\right)$ for distinct values of p and $\theta_{1}$.

The simulation of the model also assesses the combined impact of the parameters p, which is the portion of non-hesitant individuals who are not in contact with infected individuals due to social interventions and use of face masks, and $\theta_{1}$ which is the behaviour compensation post-vaccination. Non-hesitant individuals follow social norms instead of showing leniency in their behaviour; thus, we can control the disease burden. We simulated this result in figure 10.

In figure 10, we increase the level of p and decrease the level of $\theta_{1}$ from the baseline values to analyse the impact on infected individuals. If p increases by 20% and $\theta_{1}$ decreases by 20%, then the number of infected individuals decreases by 9.9%. Similarly, if p is increased by 40% and 60%, where we simultaneously decrease $\theta_{1}$, then the infected individuals would decrease by 19.9% and 30.1%, respectively. We conclude that individuals should follow social norms before and after vaccination to reduce the disease burden because after vaccination, leniency in their behaviour can increase the disease burden.

### 5.6. Impact of the vaccine efficacy $e_{1}$ and $e_{2}$ on daily number of cases

**Figure 11.**
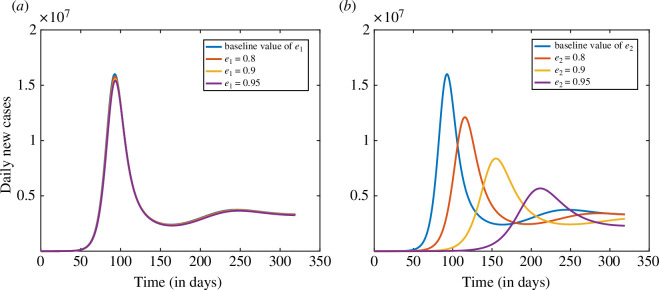
Assessing the impact of vaccine efficacies on the daily number of cases: (*a*) the vaccine efficacy $e_{1}$ and (*b*) the vaccine efficacy $e_{2}$.

The number of cases of a disease is influenced by various factors, including the efficacy of the vaccine against the disease and the level of vaccine hesitancy among the population. Vaccine efficacy refers to the ability of a vaccine to prevent disease onset or reduce the severity of symptoms in individuals receiving it. In the present study, we incorporated two types of vaccine efficacies, $e_{1}$ (the vaccine efficacy that reduces the susceptibility of vaccinated individuals) and $e_{2}$ (that reduces the duration of infection). Here, we assess the impact of the vaccine efficacies $e_{1}$ and $e_{2}$ on the daily number of cases shown in figure 11. The baseline values of the vaccine efficacy ($e_{1}$ and $e_{2}$) are 70% and 60%, respectively.

From figure 11*a*, we observe that if the vaccine efficacy $e_{1}$ is 80%, then the daily cases would be reduced by up to 1.39% from the baseline value. If the vaccine efficacy $e_{1}$ is 90% and 95%, then the daily new cases of COVID-19 would decrease by 2.85% and 3.62%, respectively, from the baseline value. Figure 11*b* depicts the impact of vaccine efficacy $e_{2}$ on daily new cases of COVID-19. If vaccine efficacy $e_{2}$ is 80%, the daily cases would decrease by up to 24.3% from the baseline value of $e_{2}$. If the vaccine efficacy $e_{2}$ is 90% and 95%, then the daily cases would be reduced by 47.6% and 64.5%, respectively, from the baseline value.

In summary, we can say that vaccine efficacy that reduces the duration of infection ($e_{2}$) has a greater impact than vaccine efficacy that reduces susceptibility ($e_{1}$). In the case of COVID-19, individuals are infected even after vaccination. Therefore, a vaccine should be developed in such a way that it also reduces the duration of infection and, hence, the disease burden.

### 5.7. Combined impact of vaccination rate δ and vaccine efficacy $e_{1}$ on infection

**Figure 12.**
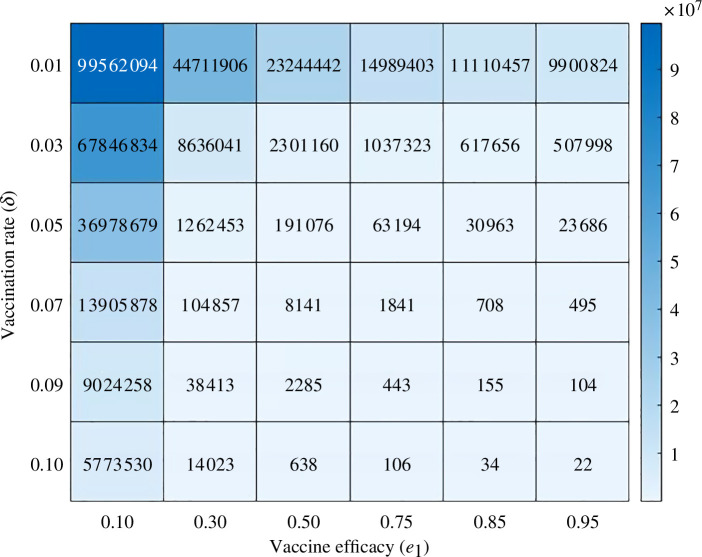
Heat map of infected individuals $\left(I_{H}+I_{NH}\right)$ for distinct values of δ and $e_{1}$.

Figure 12 presents a heat map illustrating the interaction between vaccination rates (δ) and vaccine efficacy $\left(e_{1}\right)$ on the number of infected individuals. The rows of the map represent various vaccination rates, beginning at 0.01 and increasing incrementally up to 0.10, while the columns signify vaccine efficacy, spanning from 0.10 to 0.95. The numerical values within each cell denote the number of infected individuals, with the colour gradient providing a visual representation of these figures’ magnitudes. At the lowest vaccination rate of 0.01 and the lowest vaccine efficacy of 0.10, the number of infected individuals is extremely high, estimated at approximately 99 562 094. However, as vaccine efficacy improves to 0.95 while maintaining the same vaccination rate, the number of infected individuals significantly reduces to approximately 9 900 824. This trend holds true for all vaccination rates, as higher vaccine efficacy consistently leads to fewer infections. As the vaccination rate increases to 0.10, even at the lowest vaccine efficacy of 0.10, the number of infected individuals decreases significantly to 5 773 530. With a vaccine efficacy of 0.95 at the same vaccination rate of 0.1, the number of infections further declines to just 22, demonstrating near elimination of the disease.

The heat map clearly shows how increasing both vaccination rates and vaccine effectiveness can reduce the number of infections. Higher vaccination rates lead to fewer infections, regardless of how effective the vaccine is. Similarly, a more effective vaccine results in fewer infections at any given vaccination rate. The darkest areas on the map, which indicate the highest number of infections, are found where both vaccination rates and vaccine effectiveness are low. By contrast, the lightest areas, showing the lowest infection counts, are where both vaccination rates and vaccine effectiveness are high. This visualization underscores the importance of both high vaccination coverage and effective vaccines in controlling and possibly eliminating infectious diseases.

## 6. Discussion and conclusion

Vaccination has been a beacon of hope for the fight against infectious diseases throughout history [2]. However, currently, it is increasingly difficult to sustain public trust in vaccination. In India, the COVID-19 vaccination campaign began on 16 January 2021 [55]. Despite this milestone, vaccine hesitancy has emerged as a barrier to widespread immunization. This hesitancy occurred due to misinformation and a lack of accurate information regarding vaccine safety and effectiveness. This has led to low vaccine uptake and hindered the country’s efforts to control the spread of the virus. Vaccine hesitancy has a significant impact on efforts to control the spread of COVID-19. When a large portion of the population is not vaccinated, it creates pockets of unvaccinated individuals who can still get infected and spread the virus. This undermines the concept of herd immunity, which is crucial for stopping the spread of the disease. Additionally, hesitancy regarding vaccines slows down the overall effort to eliminate the pandemic, leading to repercussions for public health, the economy and society. Therefore, it is imperative to address vaccine hesitancy to effectively control the pandemic and promote a safe lifestyle.

Several researchers have investigated COVID-19 vaccine hesitancy, with the majority of modelling studies relying exclusively on statistical models or machine learning techniques utilizing survey data [21–25]. However, there is a paucity of studies that mathematically integrate vaccine hesitancy into disease dynamics. A mathematical model plays a crucial role in analysing infectious diseases affecting humans, such as COVID-19. Through the use of mathematical models, researchers can enhance their understanding of disease transmission and progression. Moreover, these models offer critical insights for public health policymakers, enabling them to make informed decisions to control and prevent the spread of the disease. By considering these facts, we developed a compartmental model to study the impact of hesitancy and behaviour compensation on the COVID-19 infection. We divide the population of India into two subgroups: those who are hesitant towards vaccination and those who are non-hesitant towards vaccination. We consider seven compartments in this study: hesitant susceptible $\left(S_{H}\right)$, non-hesitant susceptible $\left(S_{NH}\right)$, hesitant infected $\left(I_{H}\right)$, non-hesitant infected $\left(I_{NH}\right)$, non-hesitant vaccinated $\left(V_{NH}\right)$, hesitant recovered $\left(R_{H}\right)$, non-hesitant recovered $\left(R_{NH}\right)$. Both social interventions and behaviour compensation have been incorporated to study their impacts on the COVID-19 disease.

In our model formulation, we assumed that the individuals completed the second vaccine dose of COVID-19, which was started from 13 February 2021, in India. Therefore, we fitted and parametrized the model using the daily new cases of COVID-19 from India starting from the time period when the second dose of vaccine was started in India (that is, 13 February 2021) until 12 January 2022. The model has been fitted by dividing the time period, according to the increment and decrement in the number of cases. The data have been collected from the official website of the WHO.

First, we computed the expression for the reproduction number $R_{0}$, a threshold which measures the severity of the infectious disease and prove that the disease free equilibrium is globally stable for $R_{0}<1$. Further, we computed the final size of the epidemic. The computation showed that for the period from 13 February 2021 to 8 May 2021, the value of $R_{0}$ is 3.36 when the cases were continuously increasing, for the period from 9 May 2021 to 28 December 2021 the value of $R_{0}$ is 1.22 when the cases of COVID-19 are decreasing, for the period from 29 December 2021 to 12 January 2022 the value of $R_{0}$ is 1.97 when the cases are increasing. Next, we assessed the impact of different parameters on $R_{0}$ using the surface plots in figure 6. The results shows that the reproduction number can be reduced by increasing the use of social interventions and simultaneously by reducing the ratio of hesitant susceptible.

**Figure 13.**
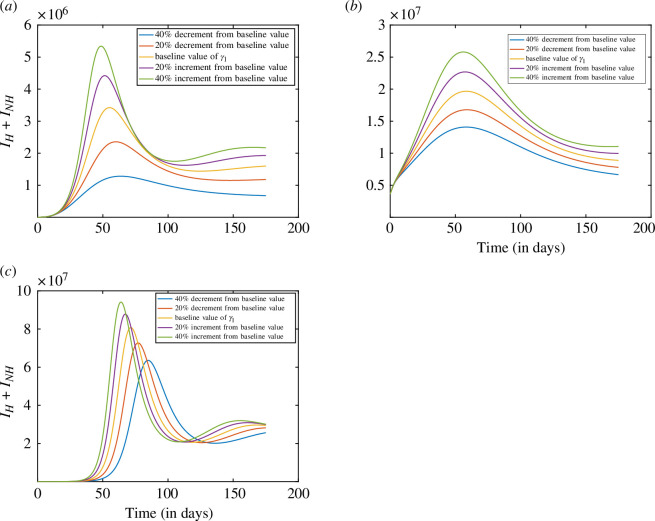
The impact of the hesitancy parameter $\gamma_{1}$ on infected individuals.

**Figure 14.**
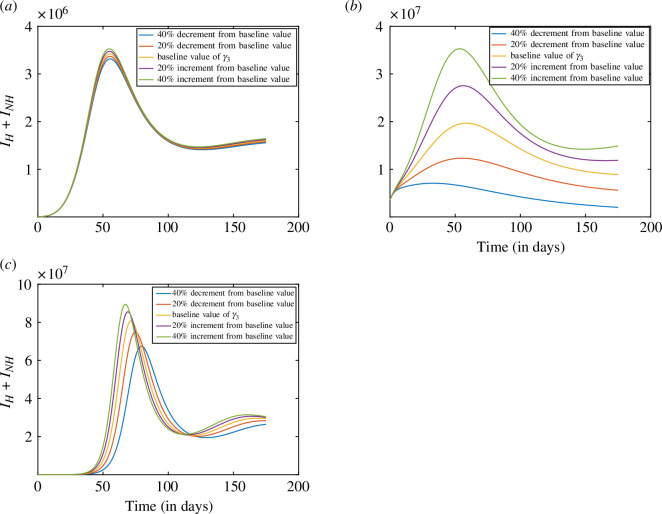
The impact of the hesitancy parameter $\gamma_{3}$ on infected individuals $\left(I_{H}+I_{NH}\right)$.

Further, we assessed the impact of the hesitancy on the infected cases of COVID-19 in India for different periods by using the parameter values from tables 2 and 3. We simulate the impact of the hesitancy which occurs in susceptible individuals $\gamma_{1}$. The result shows that if hesitancy has been continued then the number of infected individuals of COVID-19 would be increased up to 62.5%. The simulation results shown in figure 13 indicate that the susceptible individuals who are eligible for the vaccine shots should take it on time to reduce the disease burden. Next, we assessed the impact of hesitancy among infected individuals $\gamma_{3}$. This result is simulated in figure 14 for the different periods which shows that if the hesitancy parameter $\gamma_{3}$ would increase then the COVID-19 cases would also increase by 79.4% from 9 May 2021 to 28 December 2021 when the cases were at lowest. This means infected individuals would be better to take vaccine shot rather then rely on their natural attained immunity in any scenario.

The two parameters which are very crucial for reducing the disease burden are the vaccination rate and hesitancy rate. In figure 9, we increased the vaccination rate and decreased the hesitancy parameter by the same levels, i.e 20%, 40% and 60%. The result shows that if hesitancy would be decreased by 60% then the cases would be decreased by 73.2%. The next combination of the parameters are the portion of non-hesitant individuals who are not contacting with the infected individuals due to social interventions and use of face masks p and behaviour compensation post-vaccination $\theta_{1}$. We simulate the result for this combination by increasing p and decreasing $\theta_{1}$ by the same levels, i.e. 20%, 40% and 60%. The result shows that if $\theta_{1}$ would be decreased by 60% with an increase in the value of p by 60%, then infection would be decreased by 30.1%.

Further, we assessed the impact of vaccine efficacies $e_{1}$ (reduces the susceptibility to acquire infection) and $e_{2}$ (reduces the duration of infection) on the daily number of cases. The baseline values of $e_{1}$ and $e_{2}$ are 70% and 60%, respectively. If both the efficacies $e_{1}$ and $e_{2}$ are 95% then the daily cases can be reduced by 3.62% and 64.5%, respectively. From this result, we can say that the vaccine efficacy $e_{2}$ is more impactful to reduce the disease burden. Therefore, we conclude that vaccines should be developed in such a way that also reduces the duration of infection to reduce the disease burden. Next, we analyse the combined influence of vaccine efficacy $\left(e_{1}\right)$ and vaccination rate (δ) by creating a heatmap to visualize their impact on infection. Our findings suggest that a higher vaccine efficacy, coupled with an elevated vaccination rate, is crucial for reducing the overall disease burden.

Our study on the dynamics of COVID-19 vaccination in India emphasizes the significance of addressing vaccine hesitancy, ensuring post-recovery vaccination and maintaining social interventions to effectively reduce and control COVID-19 cases. When compared with other studies, our findings align with similar themes but also offer distinct insights. Buonomo *et al*. [56] introduced a compartmental model that accounts for vaccine hesitancy and the influence of information dissemination. They highlighted that voluntary vaccination reduces the disease’s impact but cannot eliminate it, emphasizing the role of widespread information and short-term memory. Khan *et al*. [57] incorporated multiple strains and vaccine options, considering waning immunity and the decision-making process based on vaccine cost-effectiveness. This study underscores the complexities of vaccine choice and the rapid adoption of vaccines with higher efficacy in the presence of more contagious strains. Along with this, these studies complement our findings by reinforcing the importance of behavioural factors in vaccine uptake and disease control, while also expanding the discussion to include information dynamics, multiple vaccine choices and waning immunity. Furthermore, research conducted by Ioannidis [58] highlights the significant impact of increased exposure risk following vaccination on the erosion of vaccine benefits, particularly when vaccine efficacy is moderate. Similarly, our study emphasizes the importance of maintaining preventive measures to control the spread of COVID-19, as relaxed behaviour post-vaccination can lead to a rise in cases. Both studies concur that risk compensation and behaviour changes after vaccination are essential factors in determining the overall success of COVID-19 vaccination initiatives. By comparing our results with these studies, we reinforce the broader understanding that addressing behavioural factors, such as vaccine hesitancy and post-vaccination behaviour, is crucial for effective disease control. Our study contributes to this body of literature by providing specific quantifications of the reduction in cases due to vaccination and the critical role of combining vaccination with social interventions.

The present study underscores the need for policymakers to prioritize and vigorously advocate for extensive vaccination campaigns as a vital strategy for mitigating the effects of infectious diseases. It is essential to select vaccines with high efficacy to achieve a swift and effective decrease in the overall disease burden. Additionally, it is crucial to disseminate information to the public, emphasizing that eligibility for vaccination extends beyond merely susceptible individuals to include those who have previously been infected and recovered. By encouraging individuals post-recovery to receive the vaccine, we can enhance overall immunity levels within the population, further contributing to the reduction of disease transmission. Clear and targeted communication strategies must be prioritized by policymakers to ensure that this critical information reaches all segments of the population, fostering a comprehensive and inclusive approach to vaccination efforts.

Our study has several limitations that should be considered when interpreting the findings. Firstly, the model is specifically tailored to COVID-19 and its findings may not be directly applicable to other infectious diseases with different transmission routes such as vectorborne or foodborne diseases. The model is calibrated with data specific to India, which may limit the generalizability of the results to other countries with varying demographic, social and healthcare contexts. Furthermore, while the study acknowledges the impact of vaccine hesitancy, it may lack detailed data on the specific factors contributing to hesitancy and their variations across different demographics and regions within India. Additionally, the model assumes a certain level of vaccine efficacy, not accounting for diseases with vaccines requiring frequent updates, such as influenza. It does not consider the impact of multiple SARS-CoV-2 variants with varying transmissibility and immune escape potential. In addition, this particular study does not address the heterogeneous mixing of populations or age-specific differences in susceptibility, transmission and vaccine response. By acknowledging these limitations, we highlight areas for future research to enhance the robustness and applicability of pandemic response models.

## Data Availability

The data used in this work are available from the COVID-19 Dashboard of the World Health Organization [59].

## Appendix A

### A.1. Proof of theorem 3.1

To prove the global stability of $E_{0}$, we use the approach given in Castilo-Chavez *et al*. [60]. We rewrite the system (2.2) as follows:

$$\begin{array}{rl} \frac{dX}{dt} & =F(X,Y),\\ \frac{dY}{dt} & =G(X,Y),\;\text{with}\;G(X,0)=0, \end{array}$$

where $X=(S_{H},S_{NH},V_{NH},R_{H},R_{NH})\in R^{5}$ (uninfected compartments), $Y=(I_{H},I_{NH})\in R^{2}$ (infected compartments). Now F(X,Y) and G(X,Y) from system (2.2) are given as follows:

$$\begin{array}{c} F(X,Y)=\left[\begin{array}{c} -\lambda S_{H}-\gamma_{2}S_{H}+\omega_{1}R_{H}+\gamma_{1}S_{NH}\\ -\delta S_{NH}-\lambda (1-p)S_{NH}-\gamma_{1}S_{NH}+\gamma_{2}S_{H}+\sigma_{3}V_{NH}+\omega_{2}R_{NH}\\ -\lambda^{V}(1-p)V_{NH}+\delta S_{NH}-\sigma_{3}V_{NH}\\ \sigma_{1}I_{H}-\omega_{1}R_{H}+\gamma_{4}R_{NH}\\ \frac{\eta }{\left(1-e_{2}\right)}I_{NH}-\left(\omega_{2}+\gamma_{4}\right)R_{NH} \end{array}\right], \end{array}$$

and

$$\begin{array}{c} G(X,Y)=\left[\begin{array}{c} \lambda S_{H}-\sigma_{1}I_{H}-\mu_{1}I_{H}+\gamma_{3}I_{NH}\\ \lambda (1-p)S_{NH}+\lambda^{V}(1-p)V_{NH}-\left(\frac{\eta }{\left(1-e_{2}\right)}+\mu_{2}+\gamma_{3}\right)I_{NH} \end{array}\right]. \end{array}$$

The global stability of the $E_{0}$ is guaranteed if the following two conditions are satisfied:

(H1) For $\frac{dX}{dt}=F(X,0)$, $X^{*}$ is globally asymptotically stable.

(H2) $G(X,Y)=BY-\hat{G}(X,Y),\;\;\hat{G}(X,Y)\geq 0$, for (X,Y)∈∧.

In this case, $B=D_{Y}G(X^{*},0)$ is an M-matrix. For the model system (2.2), we have

$$\begin{array}{c} F(X,0)=\left[\begin{array}{c} -\gamma_{2}S_{H}+\gamma_{1}S_{NH}+\omega_{1}R_{H}\\ -\delta S_{NH}-\gamma_{1}S_{NH}+\gamma_{2}S_{H}+\sigma_{3}V_{NH}+\omega_{2}R_{NH}\\ \delta S_{NH}-\sigma_{3}V_{NH}\\ -\omega_{1}R_{H}+\gamma_{4}R_{NH}\\ -(\omega_{2}+\gamma_{4})R_{NH} \end{array}\right]. \end{array}$$

Clearly, the system $\frac{dX}{dt}=F(X,0)$ is globally asymptotically stable around $X^{*}$ = $(S_{H}(0),$$S_{NH}(0),$0,0,$V_{NH}(0),$0,0). Moreover, from the model system (2.2), we obtain

$$B=\left[\begin{array}{cc} \frac{\beta S_{H}(0)}{N}-(\sigma_{1}+\mu_{1}) & \frac{\beta (1-p)S_{H}(0)}{N}+\gamma_{3}\\ \frac{\beta (1-p)S_{NH}(0)}{N}+\frac{\left(1-e_{1})(1+\theta_{1})(1-p)\beta V_{NH}(0\right)}{N} & \frac{\beta (1-p{)}^{2}S_{NH}(0)}{N}+\frac{\beta (1-e_{1})(1+\theta_{1})(1-p{)}^{2}V_{NH}(0)}{N}-\\ & \left(\frac{\eta }{1-e_{2}}+\mu_{2}+\gamma_{3}\right) \end{array}\right],$$

and

$$\hat{G}\,\left(X,Y\right)=\left[\begin{array}{c} K_{1}\\ K_{2} \end{array}\right],$$

where

$$\begin{array}{rl} K_{1} & =\frac{\beta I_{H}(S_{H}(0)-S_{H})}{N}+\frac{\beta (1-p)I_{NH}(S_{H}(0)-S_{H})}{N},\\ K_{2} & =\frac{\beta I_{H}(1-p)(S_{NH}(0)-S_{NH})}{N}+\frac{\beta (1-p{)}^{2}I_{NH}(S_{NH}(0)-S_{NH})}{N}\\ & +\frac{\left(1-e_{1})(1+\theta_{1})(1-p)\beta I_{H}(V_{NH}(0)-V_{NH}\right)}{N}\\ & +\frac{\beta (1-e_{1})(1+\theta_{1})(1-p{)}^{2}I_{NH}(V_{NH}(0)-V_{NH})}{N}. \end{array}$$

Clearly $\hat{G}(X,Y)\geq 0$. Hence, both conditions (H1) and (H2) are fulfilled. As a result, the DFE $E_{0}$ of the system (2.2) is globally asymptotically stable when $R_{0}\leq 1$.

### A.2. Impact of $\gamma_{1}$ on infected individuals

See figure 13.

### A.3. Impact of $\gamma_{3}$ on infected individuals $\left(I_{H}+I_{NH}\right)$

See figure 14.
